# A Scoping Review of Patient Health-Related Quality of Life Following Surgery or Molecular Testing for Individuals with Indeterminate Thyroid Nodules

**DOI:** 10.3390/healthcare12202025

**Published:** 2024-10-11

**Authors:** Khadija Brouillette, Raisa Chowdhury, Kayla E. Payne, Marc Philippe Pusztaszeri, Véronique-Isabelle Forest

**Affiliations:** 1 Faculty of Medicine and Health Sciences, McGill University, Montreal, QC H4A 3T2, Canada; 2 Faculty of Arts, McGill University, Montreal, QC H4A 3J1, Canada; 3 Department of Pathology, McGill University, Montreal, QC H3A 2B4, Canada; 4 Department of Otolaryngology-Head and Neck Surgery, McGill University, Montreal, QC H3T 1E2, Canada

**Keywords:** thyroid neoplasms, thyroid nodule management, molecular testing, diagnostic testing, quality of life

## Abstract

Background: Molecular testing can reduce the need for diagnostic thyroidectomy in cytologically indeterminate thyroid nodules. However, the health-related quality of life in patients managed with molecular testing is not well studied. Objective: The objective of this scoping review was to identify and analyze the health-related quality of life outcomes in patients with indeterminate thyroid nodules who are expected to undergo or have undergone surgery or molecular testing. Methods: A comprehensive search was conducted on PubMed, Scopus, PsychINFO, and Embase to identify relevant studies. The search terms included “thyroid neoplasms” or “thyroid nodule” and “molecular testing” or “surgery” and “quality of life”. The included articles were analyzed for their main study objective, study design, participant characteristics, and main results. Results: Eight studies were included in this scoping review. Four evaluated the quality-adjusted life years for patients with indeterminate thyroid nodules. Three of these studies found that molecular testing slightly improved quality-adjusted life years compared to surgery, while one study found no difference. Two studies assessed surgical health-related quality of life outcomes and reported that patients with indeterminate thyroid nodules who were expected to undergo surgery favored surgical procedures, while those who underwent surgery experienced impaired health-related quality of life. Two studies evaluated molecular testing in patients with indeterminate thyroid nodules and found that the final molecular test result significantly impacted health-related quality of life outcomes. Patients with suspicious/positive molecular test results had worse symptoms of goiter, anxiety, and depression, while those with benign results had preserved health-related quality of life scores. Patients with benign results from molecular testing experience better health-related quality of life within the first year compared to those with benign surgical outcomes. Conclusions: This scoping review highlights the importance of considering health-related quality of life outcomes in the management of patients with indeterminate thyroid nodules. Benign molecular testing results yield better quality of life than benign surgical outcomes within the first year, suggesting molecular testing as a preferable option. Further research comparing the impact of surgery and molecular testing on health-related quality of life is needed to improve shared decision-making and patient outcomes.

## 1. Introduction

The emergence of advanced medical imaging has led to the increased identification of thyroid nodules [1]. Upon identification, diagnosis and risk stratification are necessary using the gold standard, ultrasound-guided fine-needle aspirations (USFNA) [1,2]. The clinical conundrum presents as approximately 20–30% USFNAs are categorized as indeterminate thyroid nodules (ITNs) category Bethesda III (atypia (or follicular lesion) of undetermined significance) or Bethesda IV (follicular neoplasm/suspicious for follicular neoplasm) based on the 2017 Bethesda System for Reporting thyroid cytopathology [3,4]. An ITNs management plan includes diagnostic surgery consisting of a lobectomy (hemithyroidectomy) and/or a near-total thyroidectomy, with the removal of most thyroid tissue, and/or a total thyroidectomy, with the removal of all thyroid tissue [5]. ITNs have a malignancy rate between 10 and 40% [6]; thus, patients with ITNs often undergo unnecessary surgery [6,7]. Molecular testing (MT) has been an essential tool in enhancing the accuracy of diagnoses, directing effective management plans, and reducing the likelihood of unwarranted surgical procedures for patients with ITNs [5,7,8].

Molecular testing has emerged as one of the useful diagnostic tools in the management of ITNs by offering a non-invasive means to increase diagnostic yield and treatment decisions. Examples of common molecular tests include gene expression classifiers (Afirma); ThyroSeq assesses both gene mutations and expression patterns to stratify malignancy risk and mutation panels that detect the presence of specific genetic mutations associated with malignancy [9]. These tests help in differentiating benign nodules from malignant ones and avoid unnecessary surgery of the thyroid. Molecular testing facilitates shared decision-making between the patients and clinicians with its risk stratification and helps in putting forward an evidence-based treatment option according to the patient’s risk profile and preference [10]. There are currently three commercially available MTs in North America for thyroid nodules: ThyroSeq v3, Afirma, and ThyGenX/ThyraMIR. These tests have similar statistical performance in Bethesda III-IV cases, with a sensitivity of 82% to 91%, a specificity 64% to 91%, a negative predictive value 91% to 98%, and a positive predictive value 51% to 82% [8]. Even though each MT has distinct advantages and disadvantages, the literature reports a steadily rising performance and predicted nature in ITNs evaluations [8]. Thus, in patients with ITNs, a critical decision pendulum of surgery or MT becomes emphasized. These patients with ITNs face a choice between surgical procedures, which again pose some risks including scarring, hypoparathyroidism, and recurrent laryngeal nerve injury, and MT as a less invasive approach. A systematic review by Ngo et al. (2020) [11] demonstrated that MT has significantly reduced the rates of unnecessary thyroidectomies, particularly in Western countries where the availability of these molecular tests is widespread.

An optimal management plan for ITNs has not yet been clearly outlined; thus, according to the shared decision-making (SDM) model, patients with ITNs are in a state of clinical uncertainty [12]. Through SDM, patients and physicians must discuss which intervention aligns best with the patients’ values and preferences. An important consideration for shared decision-making is health-related quality of life (HrQOL) outcomes [12]. HrQOL is a subjective, multidimensional model allowing researchers to understand the physical, social, psychological, and spiritual factors influencing a patients’ quality of life (QOL) [13]. In the management of ITN, surgery and MT have different impacts on HrQOL. Surgical treatment, especially total thyroidectomy, can cause physical complications such as scarring, hypoparathyroidism, and voice changes that may significantly affect HrQOL [6,14,15]. On the other hand, although MT represents a less invasive diagnostic option for patients, suspicious molecular test results may lead to increased anxiety and depression due to uncertainty regarding malignancy [15,16]. Thus, HrQOL is an important consideration not only in clinical outcomes but also in patient-centered decision-making, whereby the clinician can align treatment options with the preferences of the patients [13].

To date, there are few studies reporting the HrQOL outcomes of surgery and MT in patients with ITNs. A narrative review describes the findings of two articles, included in this review, reporting QOL associated with MT for ITNs [17]. To comprehensively assess the coverage of current literature, it was determined that an exploratory form of methodology was required. This scoping review aimed to identify and analyze health-related quality of life (HrQOL) outcomes in patients with indeterminate thyroid nodules (ITNs) managed with molecular testing or surgery.

## 2. Materials and Methods

The Preferred Reporting Items for Systematic reviews and Meta-Analyses extension for Scoping Reviews (PRISMA-ScR) were followed to present the scoping review. The protocol for this review was retrospectively registered with PROSPERO (International Prospective Register of Systematic Reviews) under the registration number CRD42023439964. The original study was a systematic review; after a thorough examination of all the included articles, it became evident that the study’s actual objective was to identify, rather than compare, all HrQOL outcomes associated with the population and intervention. Thus, the decision was made to transform the study into a scoping review.

The inclusion criteria for this review were original studies involving human participants with indeterminate thyroid nodules (ITNs), either undergoing surgery or molecular testing (MT), that reported on health-related quality of life (HrQOL) outcomes. The review included adult populations (aged ≥18) with or without common comorbidities that could affect HrQOL, such as cardiovascular diseases (e.g., hypertension, ischemic heart disease), diabetes mellitus, and autoimmune thyroid disorders (e.g., Hashimoto’s thyroiditis). Pediatric populations, studies that did not report primary data, and studies focusing solely on cancer-related HrQOL without reference to ITNs were excluded. A search with no language or time restrictions was conducted on 15 June 2023, including the following databases: PubMed, Scopus, PsychINFO, and Embase. The search was performed in the listed database with a combination of the following search items (“thyroid neoplasms” or “thyroid nodule”) AND (“molecular testing” or “surgery”) AND (“quality of life”). Studies were included if they met the following criteria: (1) original investigations; (2) English language; (3) primary research articles; (4) studies reporting QOL outcomes including and not limited to the following domains: social, mental, financial, and functional well-being; (5) studies reporting the actual and/or potential surgical outcomes after ITNs and/or studies reporting the actual and/or potential MT outcomes after ITNs. Studies were excluded if they (1) were not an original investigation (e.g., review or meta-analysis) or had (2) absent ITNs.

**Figure 1:**
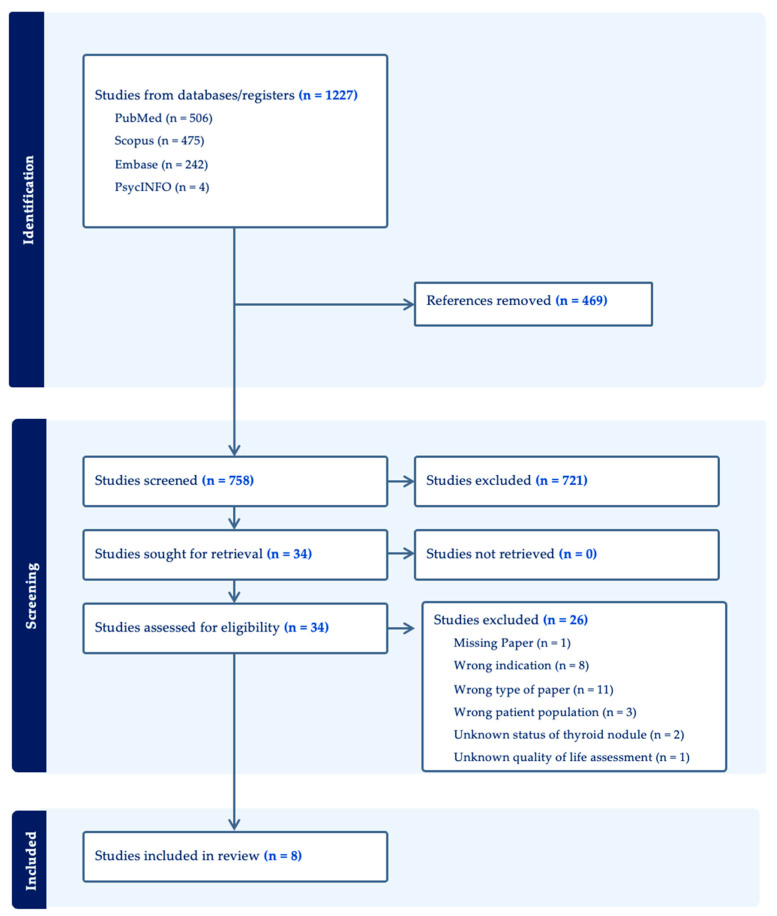
PRISMA flow diagram exported from Covidence.

The references were assessed by two blinded raters (KB and RC) who independently determined if studies met inclusion criteria on Covidence. The raters began with blinded title and abstract screening, followed by full-text article review for inclusion. All conflicts were resolved by consensus among the raters. Full search strategy details are highlighted in the PRISMA flow diagram (Figure 1). Data on patients with ITNs were extracted by the two researchers into an Excel document. The corresponding authors of four of the articles were contacted, but three did not respond, leading to their exclusion. One author provided filtered data on patients with ITNs and mean (SD) QOL scores. Extracted data included the main study objective, study design, participant characteristics, and main results. The critical appraisal of all included studies can be downloaded from Supplementary Materials (Table S1: Critical Appraisal). 

## 3. Result

This scoping review identified eight articles reporting the HrQOL outcomes in patients with ITNs who are expected to undergo or have undergone surgery or MT (Table 1).

The eight studies included in the scoping review were conducted in the United States (n = 4), Canada (n = 2), the Netherlands (n = 1), and Israel (n = 1) [15,16,18,19,20,21,22,23]. In all of the studies, participants were diagnosed or modeled to have cytologically ITNs. Regarding the HrQOL outcomes assessed for patients with ITNs, four studies evaluated the quality-adjusted life years (QALYs); two studies, qualitative and quantitative, reported surgical HrQOL outcomes; and two studies reported MT HrQOL outcomes (Table 1).

We found four studies assessing the QALYs of patients with ITNs that have undergone either surgery or MT. QALYs is a measure of the expected years of life corrected for the negative impact of diseases and disabilities on the QOL [24], with the assumption that 1 QALYs is a year lived in perfect health [24]. The included studies in the review used several methods to perform statistical analysis and the mathematical modeling of outcomes. For instance, Lee et al. [9] conducted a cost-effectiveness analysis by applying a microsimulation model with the purpose of simulating patient pathways and QALY outcomes. In contrast, Najafzadeh et al. [19] have conducted discrete event simulation modeling to project health outcomes over a 10-year time horizon 1. Thirdly, HrQOL outcomes of clinical studies were usually analyzed through the implementation of multivariate logistic regression models in view to adjust for covariates, for example, age, gender, and malignancy status. According to Pitt et al. [20], multivariate logistic regression was used to adjust for confounding factors (e.g., age, malignancy) when analyzing differences in HrQOL between molecular testing and surgical outcomes. Basic differences in methodology may have differentially affected the QALYs that could be measured across studies. In this regard, several assumptions and time horizons were generally adopted from one study to another. Despite such variations, all studies consistently showed that molecular testing modestly improved QALYs compared to current practice or surgery. In the surgical studies, patients were followed up at various times postoperatively. For example, Tzelnick et al., 2023 [22] measured HrQOL at 6 months post-surgery and Pitt et al., 2021 [20] at 12 months post-surgery. These time points are indicated in Table 1, as they may influence the outcomes measured. Overall, three studies reported that MT modestly improved QALYs for patients with ITNs compared to surgical procedures, in which two studies found that patients with ITNs using MT had slightly better QALYs compared to surgery in a simulated five and 10 year model, respectively [18,19]. Furthermore, Vriens D et al. [21] demonstrated patients with ITNs that undergo MT using gene expression classifiers (GEC) and particularly mutation marker panels had elevated QALYs compared to diagnostic thyroid surgery in a five-year simulated model. On the contrary, Lee L et al. found no difference in QALYs from a Canadian and United States perspective in MT by using GEC and a gene mutation panel when compared to a diagnostic lobectomy [9]. 

In this review, two studies reporting patients with ITN that are expected to or have undergone surgery were included. We observed that patients with ITNs expecting to undergo surgery reported favoring responses towards surgical procedures. One qualitative article reported the emotional and attitudinal responses of patients with ITNs expecting to undergo surgery [20]. Major themes in support of surgery were described, such as patients expressing a desire for reassurance that the cancer can be removed (“Get it out”), but they also experienced anxiety and fear when facing the possibility of having cancer (“It’s cancer”). They are concerned that you “can’t watch cancer: it could be spreading”, believing that “surgery [could] fix the problem”. As a result, patients minimized the likelihood and severity of any potential adverse outcomes. Tzelnick S et al. reported the QOL outcomes of patients with ITNs (n = 32) after undergoing surgery using the ThyPRO (Thyroid Patient Reported Outcome) 85-items questionnaire. ThyPRO assesses QOL (i.e., social, mental, and functional well-being), with higher scores indicating more symptoms and worse QOL [25]. Notably. patients reported elevated scores in the following domains of tiredness, emotion, and anxiety.

This review included two studies that evaluated the HrQOL among patients with ITNs who underwent MT. Both studies utilized the well-validated 39-item Thyroid-Related Patient Reported Outcome questionnaire (ThyPRO-39). In 2021, Schumm MA et al. measured the longitudinal (i.e., baseline, early, late) QOL of patients with ITNs (n = 174) that underwent MT and received a benign or suspicious result [15]. The findings revealed that patients with suspicious MT results had more pronounced symptoms of goiter, anxiety, and depression in comparison to those with benign outcomes. Additionally, individuals who obtained benign MT results maintained consistent QOL scores across all observed time intervals. However, suspicious molecular test patients who underwent definitive surgery reported improvement in nearly all ThyPro-39 QOL domains, in relation to QOL at the time of molecular test diagnosis. Similarly, in 2020, Wong CW et al. compared fine-needle aspiration biopsy (FNAB) cytology results with MT outcomes in patients with ITNs (n = 58) [16]. The study highlighted those individuals with a malignant FNAB cytology that reported greater impairment in their daily lives when compared to patients displaying suspicious MT results. Notably, patients with benign MT outcomes displayed ThyPRO-39 scores consistent with benign cytology.

**Table 1:** Included articles reporting the HrQOL outcomes in patients with ITNs.

Quality Adjusted Life Years (QALY) Studies													
Author, Year, Country, Journal	Methodology	Study Design	Sample Size	HrQOL Measures	QALY Calculation	Assessment Time Points	Sample Characteristics	Intervention	Aims of the Study	Outcome Measures	Important Results	General Summary	Key Findings
Lee L et al., 2014, Canada, Journal of Clinical Endocrinology and Metabolism [9]	Microsimulation model of patients for one-year length cycle.	The study utilized a microsimulation model to evaluate the cost-effectiveness of five management strategies for thyroid nodules with atypia of undetermined significance (AUS) cytology. The strategies compared included molecular testing approaches (gene expression classifiers and gene mutation panels) and standard management from both the U.S. and Canadian healthcare perspectives.	The model simulated a cohort of 1,000,000 patients with AUS thyroid nodules to assess lifetime costs and quality-adjusted life years (QALYs).	Health-related quality of life (HrQOL) was measured using QALYs, with utilities (quality of life weights) assigned to different health states based on existing literature. These values ranged from 0 (death) to 1 (perfect health) and were applied in the model to estimate the overall health outcomes of different treatment strategies.	The study used a microsimulation model to calculate QALYs, with varying assumptions depending on the strategy. For example, routine gene expression testing with selective gene mutation testing produced the highest QALYs (17.28 QALYs) compared to the standard management (17.11 QALYs. Different QALY values were assigned based on the patient’s health state, such as those with postoperative complications like recurrent laryngeal nerve (RLN) injury or hypoparathyroidism, which negatively impacted QALYs.	The model evaluated outcomes over the patients’ lifetime, with particular emphasis on QALYs measured over time based on complications and disease recurrence.	n = 1 million Age: 54.4 ± 21.3 ^b^ % Females: 81.0%	Gene expression testing performed using gene expression classifier (GEC). Gene mutation testing performed using gene mutation panel (GMP). Standard management was considered a diagnostic lobectomy.	To determine the cost-effectiveness of standard management and two diagnostic MTs, singly or in combination, for the atypia of undetermined significance (AUS) cytology of thyroid nodules.	Quality-adjusted life years.	Canadian healthcare system perspective, CAD 2013, mean QALYs (95% CI): Routine GEC = 17.25 (17.09, 17.41); Routine GEC with selective GMP = 17.28 (17.12, 17.45); Routine GMP = 17.02 (16.86, 17.19); Routine GMP with selective GEC = 17.12 (16.96, 17.29); Standard management = 17.18 (17.02, 17.35). United States third-party payer perspective, USD 2013, Mean QALYs (95% CI): Routine GEC = 17.23 (17.06, 17.39); Routine GEC with selective GMP = 17.28 (17.12, 17.45); Routine GMP = 17.12 (16.97, 17.29); Routine GMP with selective GEC: = 17.09 (16.93, 17.25); Standard management = 17.11 (16.94, 17.27).	QALYs appear to be very similar between surgery and MT, regardless of the perspective taken.	From the U.S. perspective, the strategy of routine gene expression classifier (GEC) combined with selective gene mutation panel (GMP) testing was the most cost-effective, reducing unnecessary surgeries while improving QALYs. From the Canadian perspective, the standard management strategy was more cost-effective, as the cost of molecular testing was higher compared to the costs of surgery in Canada. Sensitivity analyses showed that the cost-effectiveness results were highly sensitive to the costs of molecular tests and the probability of malignancy.
Li H et al., 2011, United States, Journal of Clinical Endocrinology and Metabolism [18]	Microsimulation model of patients for five one-year length cycles.	A Markov decision model was developed to evaluate the cost-effectiveness of using a novel molecular test (Afirma Gene Expression Classifier) for indeterminate thyroid nodules. The model analyzed a hypothetical cohort of adult patients using a societal perspective over a 5-year period.	The model used a hypothetical group of adult patients with cytologically indeterminate thyroid nodules.	Health outcomes were measured using quality-adjusted life years (QALYs), with values ranging from 0 (death) to 1 (perfect health). Utilities for different health states were estimated based on existing literature and expert opinion.	The study used microsimulation and time-trade-off methodology to estimate QALYs. The molecular test resulted in a mean QALY of 4.57, compared to 4.50 for current practice, indicating a modest improvement in quality of life. Utility values were drawn from literature and expert estimates, making direct comparability across methods possible, though sensitivity analysis highlighted that variability in some parameters, like test sensitivity, could influence results.	The model assessed outcomes over a 5-year horizon. The QALYs were evaluated annually within this period.	n = 1 million Age: Adults % Females: Not Available	MT using Afirma Gene Expression Classifier. Current practice was considered surgery (i.e., hemithyroidectomy or total thyroidectomy).	To determine the 5-year cost-effectiveness and health outcomes by comparing surgery and MT in patients with indeterminate FNAB from a societal perspective.	Quality-adjusted life years	MT: 4.57 QALY per patient Current practice: 4.50 QALY per patient	MT modestly improved QALY for patients with ITNs compared to surgery.	The molecular test resulted in the following: A total of 74% fewer surgeries for benign nodules. A mean cost saving of USD 1453 per patient compared to current practice. A modest increase in QALYs (0.07 QALYs) over 5 years compared to current practice.
Najafzadeh M et al., 2012, Canada, Value in Health [18]	Patient-level discrete event simulation for 10-year time horizon.	This study employed a patient-level discrete event simulation model to evaluate the cost-effectiveness of a molecular diagnostic test (DX) used in conjunction with fine-needle aspiration biopsy (FNAB) for thyroid nodules. The model compared the outcomes of using DX with current practice (NoDX), over a 10-year time horizon, for two simulated cohorts of 10,000 patients.	The model simulated 10,000 patients with indeterminate FNAB diagnoses to assess the clinical and economic outcomes of using molecular testing compared to current diagnostic practices	Health-related quality of life (HrQOL) was measured using quality-adjusted life years (QALYs). Utility values for different health states (e.g., surgery, radioactive iodine ablation, hypoparathyroidism, recurrent laryngeal nerve injury) were derived from the literature and applied to each patient in the simulation to calculate QALYs.	QALYs were calculated by multiplying the utility value of each health state with the time spent in that state. The study used a six-dimensional health state short form (derived from the SF-36 health survey) to assign utility weights to various outcomes, such as post-surgery recovery and cancer recurrence. The DX strategy was associated with a gain of 0.046 QALYs (95% CI: 0.019–0.078) per patient over a 10-year period, compared to the NoDX strategy.	The model assessed outcomes over a 10-year period, with QALYs and costs discounted at an annual rate of 3%.	n = 10,000 Age: Adults % Females: Not Available	New molecular diagnostic test. Current practice was considered surgery in accordance with TBSRTC guidelines.	To estimate the cost-effectiveness of using a molecular diagnostic (DX) test as an adjunct to FNAB, compared with current practice for initial indeterminate FNAB cytological nodules.	Quality-adjusted life years	MT: 46 QALY per 1000 patient treated QALY Gain = 0.046 QALY (95% CI 0.019–0.078) QALY loss = 0.266 QALY Current practice: QALY loss = 0.306 QALY	MT improved QALY for patient with ITNs compared to surgery.	The DX strategy resulted in fewer unnecessary diagnostic surgeries (false positives) compared to the NoDX strategy, reducing the number of unnecessary operations from 4407 to 323. DX also reduced the incidence of major complications, such as permanent hypoparathyroidism (129 cases with NoDX vs. 68 cases with DX) and recurrent laryngeal nerve injury (89 cases with NoDX vs. 29 cases with DX). The DX strategy was cost-saving, with a mean cost reduction of CAD 1087 per patient compared to NoDX. The QALY gain was driven by the reduction in unnecessary surgeries and related complications, as well as improved diagnostic accuracy.
Vriens D et al., 2014, The Netherlands, Journal of Clinical Endocrinology and Metabolism [21]	Microsimulation model of patients for five one-year length cycles	A Markov decision model was used to evaluate the cost-effectiveness of fluorodeoxyglucose-positron emission tomography/computed tomography (FDG-PET/CT) as a diagnostic tool for cytologically indeterminate thyroid nodules. The model compared FDG-PET/CT with diagnostic surgery in all patients and two molecular tests: the gene expression classifier (GEC) and the mutation marker panel (MMP).	The model represented adult patients with indeterminate fine-needle aspiration cytology (FNAC), specifically Bethesda categories III and IV nodules	Quality-adjusted life years (QALYs) were used to measure health-related quality of life (HrQOL) in the study. Utility values were obtained from the literature and expert panels, with adjustments for health states such as post-surgery recovery, surveillance, and complications.	QALYs were calculated over a 5-year time horizon. FDG-PET/CT provided 4.55 QALYs, which was slightly higher than surgery (4.52 QALYs) and comparable to the molecular tests. The study indicated that FDG-PET/CT prevented unnecessary surgeries, which contributed to the modest increase in QALYs.	The model assessed outcomes over a 5-year period, with costs and utilities discounted at 4.0% and 1.5%, respectively. The majority of surgeries and complications occurred within the first few years of the time horizon.	n = 1 million Age: Adults % Females: Not Available	MT using GEC and MMP. Surgery consisting of diagnostic thyroid surgery.	To determine the 5-year cost-effectiveness for FDG-PET/CT implementation in adult patients with indeterminate FNAB cytology compared with surgery and MTs.	Quality-adjusted life years	MT: GEC: 4.556 (95% CI 4.552–4.560) QALY per patient MMP: 4.515 (95% Cl 4.511–4.519) QALY per patient Surgery: 4.516 (95%CI 4.512–4.520) QALY per patient	MT slightly improved QALY for patient with ITN compared to surgery.	FDG-PET/CT resulted in 40.3% unnecessary surgeries for benign nodules, compared to 75.0% with surgery, 38.2% with GEC, and 75.0% with MMP. The FDG-PET/CT strategy was found to be the most cost-effective, with an incremental net monetary benefit (iNMB) of EUR 3684 compared to surgery, EUR 1030 compared to GEC, and EUR 3851 compared to MMP. Over 5 years, FDG-PET/CT had a mean cost of EUR 7983, which was lower than both surgery (EUR 8804) and GEC (EUR 9341).

Pitt SC et al., 2021, United States, Thyroid [20]	This qualitative study involved semistructured interviews with 85 patients diagnosed with papillary thyroid cancer (PTC) or an indeterminate thyroid nodule before undergoing thyroidectomy. The grounded theory methodology was used to develop a conceptual model of patients’ emotional and psychological responses to diagnosis.	Prospective qualitative study	A total of 85 participants were interviewed, including 50 patients with confirmed papillary thyroid cancer (PTC) and 35 patients with indeterminate thyroid nodules (Bethesda III or IV cytology).	This study focused on the emotional and psychological responses of patients, but it did not use a formal health-related quality of life (HrQOL) scale like SF-36 or ThyPRO. Instead, it explored themes like anxiety, fear, and the psychological impact of the diagnosis on patients’ quality of life.	This study did not calculate QALYs. It qualitatively explored patients’ emotional reactions to the diagnosis of thyroid cancer or indeterminate nodules, such as their immediate fear and urgency to “get it out” upon hearing the word “cancer”.	Interviews were conducted before surgery, providing insight into the participants’ emotional responses shortly after receiving their diagnosis. The interviews took place approximately 23.8 days after the initial diagnosis on average.	Total n = 85 ITN n = 35 Total Age: 48 ± 13.5 ^b^ Total % Females: 73%	Patients diagnosed with indeterminate thyroid nodules that are expected to undergo surgery within 2 months.	To understand patient experiences surrounding diagnosis of thyroid cancer or an indeterminate FNAB cytology.	Semi Structured interview	Themes identified from patients with ITNs “Get it out” represents the desire and reassurance that the potential cancer could be removed. “Its Cancer” describes the anxiety and fear in response to the potential cancer. “You can’t watch cancer: it could be spreading” showcasing fear and worry associated with potential cancer. “Surgery will fix it” suggesting removing the potential cancer would reduce negative emotions. “Minimizing the likelihood and severity of potential adverse outcomes” related to the ‘get it out’ theme whereby the potential severity of complications from surgery was lessened.	Negative emotions and the “get it out” response was observed in patients with ITN about to undergo surgery	The dominant theme among participants was a strong desire to “get it out,” referring to the urgency to remove the cancer or nodule as quickly as possible. This reaction occurred regardless of whether the participant had confirmed PTC or an indeterminate diagnosis. Many participants expressed that the word “cancer” elicited immediate fear, anxiety, and shock, which persisted even after receiving reassurance about the good prognosis of PTC. The participants’ emotional reactions often overshadowed their ability to fully process information about less extensive treatments or active surveillance.
Tzelnick S et al., 2023, Israel, Journal of Laparoendoscopic & Advanced Surgical Techniques [22]	This was a retrospective review of the database of a tertiary medical center. The study compared quality of life outcomes between two groups of patients who underwent thyroidectomy: those who underwent transaxillary robotic hemithyroidectomy and those who underwent conventional cervical surgery. The ThyPRO (Thyroid-Specific Quality of Life) questionnaire was used to measure quality of life outcomes.	Retrospective cross-sectional study	The cohort consisted of 131 patients, with 63 undergoing robotic hemithyroidectomy and 68 undergoing conventional thyroidectomy.	Health-related quality of life (HrQOL) was measured using the ThyPRO questionnaire, which assesses physical and mental health outcomes in thyroid patients. The study particularly focused on parameters such as anxiety, depression, cognitive function, and sex life impairment.	This study did not calculate QALYs. It focused on comparing postoperative quality of life outcomes between robotic and conventional thyroidectomy using the ThyPRO scores.	The specific time points for QOL assessments are not mentioned in the abstract. Full paper access may be required to clarify when the ThyPRO questionnaire was administered postoperatively.	n = 32 Age: 45 ± 16.29 ^b^ % Females: 87.5%	Surgery consisting of transaxillary robotic thyroidectomy and conventional thyroidectomy.	To compare postoperative QOL between patients that underwent transaxillary robotic thyroidectomy or conventional thyroidectomy from 2012 to 2022.	ThyPRO Quality of Life Questionnaire 85-item	Post-surgery Thy-Pro-39 result for cytologically indeterminate thyroid nodule, mean ± SD: Goiter: 9.08 ± 12.05 Hyperthyroidism: 7.03 ± 9.47 Hypothyroidism: 7.42 ± 15.18 Eye symptoms: 5.57 ± 10.68 Tiredness: 26.79 ± 18.19 Cognitive problems: 7.94 ± 16.48 Anxiety: 10.42 ± 18.60 Depression: 9.04 ± 14.94 Emotion: 15.45 ± 15.29 Impaired social life: 3.71 ± 12.29 Impaired daily life: 9.24 ± 14.35 Impaired sex life: 5.86 ± 14.65 Cosmetic complaints: 3.11 ± 7.62	Notably, patients had more symptoms of tiredness, emotion, and anxiety.	Patients who underwent robotic hemithyroidectomy reported better QOL outcomes in terms of physical and mental health, specifically experiencing decreased anxiety, depression, and cognitive and sex life impairments (*p* < 0.0001). After adjusting for factors like age, gender, malignancy status, and surgical approach, patients in the robotic group had a lower probability of experiencing depressive symptoms compared to the conventional surgery group (odds ratio = 0.31; 95% CI, 0.11–0.88). There was no significant difference in cosmetic outcomes between the two groups.
**MT Studies**													
Schumm MA et al., 2021, United States, Annals of Surgical Oncology [15]	Prospective longitudinal study.	A total of 252 patients were eligible for the study, with 174 completing the ThyPRO-39 QOL assessment. Of these, 124 had benign molecular test results and 50 had suspicious results.	The ThyPRO-39 questionnaire was used to measure health-related quality of life (HrQOL) across multiple domains, including goiter symptoms, anxiety, depression, cognitive complaints, social impairment, and emotional susceptibility.	This study did not calculate QALYs. It focused on evaluating longitudinal QOL changes in different groups (benign vs. suspicious molecular test results) using the ThyPRO-39 scale scores.	Patients were assessed at baseline (within 0–4 months post-FNA), early follow-up (4–12 months), and late follow-up (12–24 months). Postoperative assessments for suspicious molecular test patients occurred a median of 7.6 months after surgery.	n = 174 Age: 56 (44–66) ^a^ Percentage of Females: 77%	MT using either Afirma Genomic Sequence Classifier or ThyroSeq v3.	To assess the longitudinal QOL of patients who underwent FNA biopsy with indeterminate cytology (Bethesda III/IV) that are undergoing surveillance after a benign MT result or thyroidectomy after a suspicious MT result.	*ThyPRO-39* Administered at three-time points; (1) baseline (0–4 months after FNA), (2) early (4–12 months after FNA), and (3) late (12–24 months after FNA)	MT Thy-Pro-39 result for cytologically indeterminate thyroid nodule, mean ± SD: Goiter: MT benign: (Baseline: 12.0 ± 15.0 ^+^ (n = 102), Early: 11.4 ± 11.9 (n = 71), Late: 11.3 ± 13.3 (n = 52)) MT suspicious: (Baseline: 21.1 ± 23.7 (n = 45), Early: 11.1 ± 15.5′ (n = 9), Late: -- ) Anxiety: MT benign: (Baseline: 24.8 ± 17.8 ^+^ (n = 102), Early: 19.9 ± 22.1 (n = 71), Late: 20.2 ± 22.8 (n = 52) MT suspicious: (Baseline: 33.9 ± 26.6 (n = 45), Early: 24.6 ± 19.1 (n = 9), Late: --) Depression: MT benign: (Baseline: 24.0 ± 17.8 ^+^ (n = 102), Early: 21.6 ± 15.0 (n = 71), Late: 22.4 ± 17.3 (n = 52), MT suspicious: (Baseline: 36.6 ± 22.6 (n = 45), Early: 27.0 ± 15.1 (n = 9), Late: --) Patients with suspicious MTs reported worse symptoms of goiter, anxiety, and depression compared to benign MTs at baseline ( *p* < 0.05). No difference in symptoms at the early time point. Emotional susceptibility: MT benign: (Baseline: 26.3 ± 19.1 (n = 102), Early: 24.3 ± 19.3 (n = 71), Late: 25.9 ± 20.0 (n = 52)) MT suspicious: (Baseline: 31.8 ± 22.4 (n = 45), Early: 33.0 ± 18.9 (n = 9), Late: --) Impaired social life: MT benign: (Baseline: 14.5 ± 18.6 (n = 102), Early: 13.1 ± 16.5 (n = 71), Late:--) MT suspicious: (Baseline: 19.2 ± 23.0 (n = 45), Early: 11.1 ± 17.2 (n = 9), Late: --) Impaired daily life: MT benign: (Baseline: 10.5 ± 16.4 (n = 102), Early: 9.0 ± 14.8 (n = 71), Late:--) MT suspicious: Baseline: 15.4 ± 21.0 (n = 45), Early: 7.2 ± 9.2 (n = 9), Late: --) Appearance: MT benign: (Baseline: 18.7 ± 23.0 (n = 102), Early: 13.3 ± 19.6′ (n = 71), Late: 14.3 ± 17.6 (n = 52)) MT suspicious: (Baseline: 18.5 ± 21.1 (n = 45), Early: 10.9 ± 19.4 (n = 9), Late: --) Patients with benign MTs have no significant longitudinal changes in QOL outcomes. At baseline to early follow-up, but not late follow-up appearance improved (*p* = 0.048)		MT, with a benign result has preserved QOL score across all time intervals. MT, with suspicious result has worse QOL score compared to benign result.	Patients with benign molecular test results maintained stable QOL scores over 18 months of ultrasound surveillance, with no significant worsening of anxiety or depression. Patients with suspicious molecular test results who underwent thyroidectomy reported improved QOL post-surgery, particularly in symptoms of goiter, anxiety, depression, and impaired social life. At 8 months postoperatively, the QOL improvements in patients with suspicious molecular results were significant compared to their baseline pre-surgery scores.
Wong CW et al., 2020, United States, Endocrine Practice [16]	Prospective cohort study.	A total of 366 patients completed the QOL assessment, out of 825 patients who consented to the study. The final analysis included 332 patients, after excluding those who completed the survey post-surgery.	Health-related quality of life (HrQOL) was measured using the ThyPRO-39, which evaluates domains such as symptoms of goiter, anxiety, depression, and impaired daily life.	This study did not calculate QALYs. It focused on comparing QOL outcomes between patients with benign and suspicious molecular test results using the ThyPRO-39 scale.	The ThyPRO-39 QOL survey was administered after patients received their FNA and molecular test results, with a median time from FNA to survey completion of 57 days.	Total n = 332 ITN n = 58 Age: 54.9 ± 15 ^b^ % Females: 82%	MT using either Afirma Gene Expression Classifier or ThyroSeq v2.	To assess the impact of MT results on the QOL of patients with FNA indeterminate cytology (Bethesda III/IV) nodules.	ThyPRO-39	FNA cytology Thy-Pro-39 results for thyroid nodules, mean (SD): Goiter: Cytologically benign: 13.3 (17.2) Cytologically malignant: 20.6 (20.3) Anxiety: Cytologically benign: 22.5 (23.6) Cytologically malignant: 34.2 (30.5) Depression: Cytologically benign: 27.7 (20.7) Cytologically malignant: 32.1 (17.8) Impaired daily life: Cytologically benign: 12.0 (19.6) Cytologically malignant: 25.8 (28.4) Patients with malignant cytopathology compared to patients with ITN suspicious MT reported more daily life impairment (*p* = 0.003). No difference in symptoms of goiter, anxiety, or depression. MT Thy-Pro-39 result for cytologically indeterminate thyroid nodule, mean (SD): Goiter: MT benign: 10.4 (12.9) MT suspicious: 20.5 (20.8) Anxiety: MT benign: 22.8 (19.2) MT suspicious: 25.7 (27) Depression: MT benign: 21.0 (14.9) MT suspicious: 33.3 (24.7) Impaired daily life: MT benign: 11.6 (16.6) MT suspicious: 8.3 (16.6) Patients with benign MTs compared to patients with suspicious MT reported fewer symptoms of goiter (*p* = 0.033) and depression (*p* = 0.026). No significant differences in symptoms of anxiety or impaired daily life.		MT, with a benign result had consistent QOL score as FNAB cytological benign nodules. MT, with suspicious result has worse QOL score compared to benign result.	There were no significant differences in QOL between patients with benign FNA and patients with indeterminate FNA with benign molecular test results. Patients with suspicious molecular test results experienced significantly worse symptoms of goiter (20.5 vs. 10.4, *p* = 0.033) and depression (33.3 vs. 21.0, *p* = 0.026) compared to those with benign molecular test results. No significant differences were found in anxiety or impaired daily life between patients with benign and suspicious molecular test results.

Legend: MT: molecular testing, Thy-Pro-39: thyroid-related patient reported outcome 39-item version, TBSRTC: The Bethesda System for Reporting Thyroid Cytopathology, FNAB: fine-needle aspiration biopsy, GEC: gene expression classifier, GMP: gene mutation panel, MMP: mutation marker panel, FDG-PET/CT: fluorodeoxyglucose-positron emission tomography/computed tomography, n: sample size, ITN: indeterminate thyroid nodule, QOL: quality of life. ^a^ Age represented as median (interquartile range [IQR]); ^b^ age repressed by mean (standard deviation).

FNAB cytology results. Moreover, patients with benign MT results reported fewer symptoms of goiter and depression compared to their counterparts with suspicious MT outcomes.

## 4. Discussion

To our knowledge, this is the first scoping review to identify the HrQOL outcomes in patients with ITNs that are expected to or have undergone surgery or MT. The limited number of included studies highlights the novelty of the field, since much of the existing literature describes the surgical QOL outcomes in patients with malignant nodules. Importantly, the management of cytologically indeterminate thyroid nodules (CITNs) remains controversial, as their malignancy is difficult to establish, leading to uncertainties about treatment choices. Studies, including Alqahtani et al. (2023) [26], emphasize the need for personalized approaches that take into account both clinical and molecular findings to avoid overtreatment, especially for benign nodules. This supports our hypothesis that HrQOL outcomes can vary significantly by risk stratification handled in clinical practice, therefore complicating decisions for patients with ITNs even more.

We therefore attempt to contextualize the findings of this review within the existing saturated thyroid neoplasm literature [27]. Our review identified marginally improved QALYs for those undergoing MT compared to surgery [18,19,21], with the assumption that 1 QALY is a year lived in perfect health, and a value less than 1 is a year of life lived in less than perfect health [24]. The reported complications and persisting physical symptoms of surgery, such as recurrent nerve injuries, post-surgical hypoparathyroidism, pain, and paresthesia [28], may be associated with lower QALY in comparison to MT. However, these studies showed better QALYs for MT in three of the analyses, and in one there was no significant difference between MT and surgery. This incongruity is possibly a result of discrepancies in methodology in calculating QALYs, which include variation in simulation models applied, such as microsimulation versus discrete event simulation, and assumptions about patient recovery and long-term HrQOL [9,19]. Moreover, patient characteristics, like age and other comorbid conditions, can account for further factors because these may lower HrQOL for older patients or those with other health conditions, irrespective of the intervention [20]. 

Additionally, patients undergoing a thyroid neoplasm-related surgery report impairment in psychological, physical, and social HrQOL [29,30]. This is further complicated by the poorer HrQOL noted in patients with suspicious or positive molecular test results, who are often more anxious and depressed by the possibility of malignancy, which in turn may affect decision-making to overtreat or make anxiety-driven management decisions [16]. This finding raises the issue of psychological support and clear communication by the healthcare provider to help these patients navigate their options and decrease their emotional burden. Shared decision-making models, emphasizing patient education and counseling, have a potentially mitigating effect on the psychological impact [20]. According to Lewis [31], shared decision-making respects a patient’s relational autonomy since it involves recognition of the patient’s personal relationships and the general context in healthcare. Relational autonomy demonstrates the fact that the decisions of patients regarding certain medical issues arise from their social environment, while inclusion of SDM into clinical practice provides the likelihood of more personalized care [31]. It is, however, very important to ensure that SDM does not infringe on patient autonomy through coercion as over-informing or even pressuring the patients may undermine their capacity for self-determination. Furthermore, according to Montori et al. (2023) [32], SDM involves a care approach that encourages a joint conversation between patients and clinicians in co-developing a care plan which aligns with the patient’s specific needs and preferences [32]. It allows the patients and clinicians to work together to explore options in care, to match those with the values of the patient, as well as how to emotionally, practically, and intellectually cope with care. This can be particularly helpful in patients who need to make very difficult decisions regarding surgery or active surveillance and could allow them to feel supported in their decisions. The relational dynamics of SDM reduce anxiety, improve HrQOL, and ensure that patients feel empowered to make informed decisions about their treatment options.

Our review identified one study that illustrated elevated symptomology in tiredness, emotion, and anxiety in patients with ITNs that underwent surgery by virtue of a linear 0–100 scale, with higher scores indicating worse QOL [22]. Despite the reported impairment, a study noticed a positive attitude and emotional disposition towards anticipated surgery for patients with ITNs [20]. Patients with ITNs reported recurring themes of wanting to “get it out” and fear that “it’s cancer”. The label of cancer is associated with significant psychological and social distress [20]; however, when given another descriptor, patients were more likely to favor nonsurgical management plans [33]. 

Perhaps the adverse complications and impaired QOL associated with surgery are minimized when compared to the distress of a potential cancer. Thus, the contrast of HrQOL impairment and favoring attitudinal responses warrants further investigation into how patients with ITNs perceive surgery and what they experience after surgery. MT has been introduced as a valuable tool to improve the accuracy of diagnoses and reduce the likelihood of unnecessary surgery [5]. The articles included a focus on the HrQOL outcomes based on patients with ITNs MT results (i.e., benign or suspicious) [15,16]. This presents an interesting avenue for understanding HrQOL of patients with ITNs, as it appears to be dependent on the final result.

Patients with suspicious/positive MT results had worse symptoms of goiter, anxiety, and depression compared to patients with benign results [15]. However, suspicious molecular test patients who underwent definitive surgery reported improvement in nearly all ThyPro-39 QOL domains, in relation to QOL at the time of molecular test diagnosis [15]. Akin to active surveillance, it is also likely that the levels of cancer worry of patients with suspicious MT results decreases over time and that they would eventually express satisfaction with their choice of management option [34]. Additionally, patients with benign MT results had persevered with a longitudinal QOL score in comparison to FNAB benign cytology [15,16], suggesting that the potential diagnosis and treatment of thyroid cancer can have a significant impact on patients’ emotional well-being as seen in patients diagnosed with FNAB malignant cytology [35].

One Australian cohort study reported that patients undergoing a thyroidectomy for a benign or malignant disease had no overall difference in HrQOL [23]. Consequently, there appears to be a discrepancy between HrQOL outcomes and final pathology and/or results between both interventions, thus presenting a new line of questioning to be investigated. Cultural and geographical differences are likely to play a significant role in the psychological impact of a (potential) diagnosis of thyroid cancer. Furthermore, surgery can provide peace of mind to a subset of patients whose primary concern is to have the “cancer” or “potential danger removed”.

Assessing QOL differences between MT and surgery is challenging due to skewed data from suspicious MT results. Nevertheless, subdividing MT results between benign and suspicious/positive results leads to improved QOL outcomes when comparing benign MT results to benign surgical outcomes. According to Schumm’s study [15], patients with benign MT results showed significantly better QOL outcomes within the first year of management when comparing anxiety, depression, appearance, and impaired social and daily living. In this study, where higher numbers indicate worse QOL outcomes, anxiety levels for patients with benign MT results 4–12 months following the biopsy were 19.9, compared to 26.5 for those with benign surgical outcomes 5–12 months post-surgery. Depression levels were 21.6 in the benign MT group vs. 30.3 in the benign surgery group. For physical appearance, the results were 13.3 compared to 27.6, which is intuitive since surgery often leaves a scar. As a result, it seems as if the patients with a negative MT result score the highest when assessing for QOL.

In essence, the findings are relatively similar to the studies that have compared the QOL of patients with small papillary carcinomas that have undergone either active surveillance (without molecular testing) or immediate surgery [36]. The findings suggest that MT with benign results is a reasonable alternative to surgery for patients with ITNs from the point of view of QoL and psychology. In addition to molecular testing, including gene expression classifiers and mutation panels, emerging studies on microRNAs represent a promising direction in the molecular characterization of indeterminate thyroid nodules. Several studies [37,38,39] have suggested that certain miR profiles may be helpful in distinguishing benign from malignant nodules, thus offering improved diagnostic and prognostic results. Future studies are also needed to determine how incorporation into clinical practice can lead to changes in health-related quality of life and decision-making in the case of a patient with indeterminate nodules.

In clinical practice, the results of MT will help to determine whether surgical intervention is necessary. Generally, those patients with benign MT are treated conservatively with active surveillance, thereby avoiding thyroidectomy. On the other hand, suspicious or positive MT usually results in the performance of surgery due to a higher risk of malignancy. It is believed that such risk stratification allows for individualized patient care and prevents overtreatment associated with thyroid nodules of undetermined origin [16].

### Limitations

The primary limitation of this scoping review is the absence of the quality evaluation of the included articles. Given the heterogeneity of the studies and the objectives of a scoping review, quality assessment was deemed unnecessary. A secondary limitation is the exclusion of studies that reported ITNs as part of a larger dataset. Lastly, we could not include ITNs in the search criteria, as this term is not standardized across the literature.

Several key limitations exist within the current literature on HrQOL in patients with ITNs. The small sample sizes in most studies limit the statistical power of the findings, particularly in cohorts with suspicious molecular test results. The short follow-up periods also restrict the ability to assess the long-term effects of MT on HrQOL. Moreover, there is no uniformity in the HrQOL measurement tools used across different studies (e.g., ThyPRO-39, SF-36), making it difficult to compare results [16,21]. Future research should aim to bridge these gaps. Another limitation of this review is the inability to explore the impact of cultural and geographical differences on HrQOL outcomes. Cultural perceptions of illness, especially cancer, vary widely, and can influence patients’ responses to treatment decisions. Further research should investigate the impact of cultural factors on decision-making and HrQOL outcomes in patients with ITNs.

## 5. Conclusions

This scoping review highlights the particular importance of consideration of HrQOL outcomes in patients with ITNs. According to the findings, MT has the potential to significantly improve HrQOL in many of these patients, especially in those with benign test results, by avoiding unnecessary surgeries and related physical and psychological suffering. In contrast, suspicious or positive MT results could elicit more anxiety or depression in the patient; therefore, psychological support and shared decision-making processes may be needed to guide treatment decisions. These favorable HrQOL outcomes, associated with benign MT findings in the first year of management, would support the strong consideration of MT in patients with ITNs who wish to avoid the physical insults of surgery, including scarring and possible post-surgical complications. Long-term comparative studies of HrQOL after MT versus surgery are required, along with the assessment of how HrQOL changes over time in patients with suspicious MT results. Moreover, future research will be required to standardize the HrQOL measurement tool and conduct long-term follow-up studies to further elucidate the ways by which MT is optimized for improved patient outcomes in diverse populations.

## Supplementary Materials

The following supporting information can be downloaded at https://www.mdpi.com/article/10.3390/healthcare12202025/s1, Table S1: Critical Appraisal.
