# Supplementary material for: A Scoping Review of Patient Health-Related Quality of Life Following Surgery or Molecular Testing for Individuals with Indeterminate Thyroid Nodules
Source: Healthcare (Basel). 2024 Oct 11;12(20):2025. doi: 10.3390/healthcare12202025 (PMC11507389; doi:10.3390/healthcare12202025)
Supplement: Supplementary file 1 [file healthcare-12-02025-s001.zip › healthcare-3155062-supplementary.pdf]

healthcare

MDPI

Table S1. JBI Critical Appraisal

JBI Critical Appraisal (economic evaluations)

Citation (year)	**Is there a well-defined question?**	**Is there comprehensive description of alternatives?**	**Are all important and relevant costs and outcomes for each alternative identified?**	**Has clinical effectiveness been established?**	**Are costs and outcomes measured accurately?**	**Are costs and outcomes valued credibly?**	**Are costs and outcomes adjusted for differential timing?**	**Is there an incremental analysis of costs and consequences?**	**Were sensitivity analyses conducted to investigate uncertainty in estimates of cost or consequences?**	**Do study results include all issues of concern to users?**	**Are the results generalizable to the setting of interest in the review?**	**Overall appraisal:**
Li H et al., 2011 [18]	yes	unclear	yes	yes	unclear	yes	yes	yes	yes	yes	yes	include
Vriens D et al., 2014 [21]	yes	yes	yes	yes	unclear	yes	yes	yes	yes	yes	yes	include
Lee L et al., 2014 [9]	yes	unclear	yes	yes	unclear	yes	yes	yes	yes	yes	yes	include
Najafzadeh M et al., 2012 [19]	yes	unclear	yes	yes	unclear	yes	yes	yes	yes	yes	yes	Include

JBI Critical Appraisal Checklist for cohort studies

	**Were the two groups similar and recruited from the same population?**	**Were the exposures measured similarly to assign people to both exposed and unexposed groups?**	**Was the exposure measured in a valid and reliable way?**	**Were confounding factors identified?**	**Were strategies to deal with confounding factors stated?**	**Were the groups/participants free of the outcome at the start of the study (or at the moment of exposure)?**	**Were the outcomes measured in a valid and reliable way?**	**Was the follow up time reported and sufficient to be long enough for outcomes to occur?**	**Was follow up complete, and if not, were the reasons to loss to follow up described**	**Were strategies to address incomplete follow up utilized?**	**Was appropriate statistical analysis used?**	**Overall appraisal:**

									**and explored?**			
Liu Cn et al., 2022 [30]	n/a	n/a	unclear	unclear	unclear	yes	yes	yes	unclear	unclear	yes	include
Schumm MA et al., 2021inal [15]	yes	yes	unclear	unclear	yes	yes	yes	yes	unclear	unclear	yes	include
Wong CW et al., 2020 [16]	yes	yes	unclear	unclear	unclear	yes	yes	yes	unclear	unclear	yes	include e

JBI Critical Appraisal Checklist for Qualitative Research

	Is there congruity between the stated philosophical perspective and the research methodology?	Is there congruity between the research methodology and the research question or objectives?	Is there congruity between the research methodology and the methods used to collect data?	Is there congruity between the research methodology and the representation and analysis of data?	Is there congruity between the research methodology and the interpretation of results?	Is there a statement locating the researcher culturally or theoretically?	Is the influence of the researcher on the research, and vice-versa, addressed?	Are participants, and their voices, adequately represented?	Is the research ethical according to current criteria or, for recent studies, and is there evidence of ethical approval by an appropriate body?	Do the conclusions drawn in the research report flow from the analysis, or interpretation, of the data?	Overall appraisal:
Pitt SC et al., 2021 [20]	yes	yes	yes	yes	yes	no	no	yes	yes	yes	Yes
